# First molecular detection of *Leishmania (Leishmania) infantum chagasi* in a domestic cat (*Felis catus*) from an urban area in eastern Amazon

**DOI:** 10.1590/1678-9199-JVATITD-2022-0048

**Published:** 2023-05-22

**Authors:** Délia Cristina Figueira Aguiar, Daniela de Nazaré dos Santos Nascimento, Dinaiara Fragoso Penner, Brena do Socorro Lima de Castro, Rodrigo Rodrigues Virgolino, Alan Marcel Pamplona Neves, Andrei dos Santos Siqueira, Evonnildo Costa Gonçalves

**Affiliations:** 1 Institute of Biological Sciences, Federal University of Pará (UFPA), Belém, PA, Brazil.; 2 Federal Rural University of the Amazon (UFRA), Belém, PA, Brazil.

**Keywords:** Feline leishmaniasis, Leishmania (Leishmania) infantum chagasi, Molecular diagnostic, Eastern Amazon, State of Pará, Amazon region

## Abstract

**Background:**

Domestic cats have been implicated as accidental hosts of *Leishmania* sp. However, in recent years, the recurrent description of new cases in endemic and nonendemic areas draw attention to the potential epidemiological role of cats as reservoir hosts. Although dogs are considered urban reservoirs, cats could act as a secondary natural reservoirs in these areas. Thus, feline leishmaniasis has become an emerging disease in several countries worldwide.

**Case presentation:**

This study aimed to describe the first case of feline leishmaniasis in a stray animal that presented lesions compatible with the disease in Belém, Pará, Brazil, an important urban area in eastern Amazon. Serological tests for *Leishmania infantum* (ELISA and IFA) were nonreactive, whereas histopathological examination indicated infectious dermatitis caused by *Leishmania* spp. or *Toxoplasma gondii*. Cytopathological study of lesion aspirate confirmed the presence of *Leishmania* sp. amastigotes within macrophages. Finally, molecular analyses revealed that the feline infection was caused by *Leishmania* (*Leishmania*) *infantum chagasi*.

**Conclusion:**

To the best of the authors’ knowledge, this study reports the first case of natural infection by *Leishmania* (*Leishmania*) *infantum chagasi* in a feline from eastern Amazon. These findings suggest domestic cats as potential secondary reservoir hosts of *Leishmania* spp. in Belém, which reinforces the importance of further epidemiological investigation of feline leishmaniasis, especially in urban areas with human cases.

## Background

Leishmaniasis is a globally distributed and potentially fatal zoonosis caused by a parasitic protozoan of the genus *Leishmania*. Despite this, leishmaniasis is still considered a neglected disease among tropical diseases [1]. It is transmitted by sandfly vectors found in the New and Old Worlds, and dogs are considered the main domestic reservoir hosts. Canine leishmaniasis is a complex disease in which many infected dogs do not show clinical signs or only develop mild signs, thus supporting the survival of the parasite [2]. Symptomatic dogs may present a broad spectrum of clinical signs that hinder the diagnosis and management of the disease [3]. 

In addition to dogs, cats could be infected by different *Leishmania* species, but the prevalence of the disease in this domestic animal is much lower than in dogs, and most cases are asymptomatic [4]. When clinical signs occur, the most common are lymphadenomegaly; splenomegaly; weight loss; anorexia; and skin, mucocutaneous and eye lesions [5]. Some studies indicate that cats are less susceptible to the disease than dogs, and in endemic areas, the role of cats in leishmaniasis epidemiology is not yet understood [6, 7]. Other studies have indicated a dramatic increase in the number of cases of feline leishmaniasis (FeL), with the majority being *L. infantum/L. chagasi* [5, 8]. Nevertheless, the clinical diagnosis of FeL in endemic areas is not common, probably due to subclinical infection occurring in most infected cats [9]. It is believed that coinfections such as haemoparasites and some immunosuppressive viruses may affect the development of the disease [10]. 

Since cats have a freer lifestyle than dogs, and it is assumed that they would be exposed to a greater number of arthropods than dogs. It seems that the use of the tongue for hygiene by cats seems to minimize these risks compared to the risks for dogs [11]. However, in cases where there is abundance of the vector in the peridomiciliary area, infection is favored. The occurrence of FeL has been described in several countries [12-14]. In Brazil, there are reports of FeL in domestic cats in several states [8, 15, 16, 17], including Pará [18]. In the present study, we provide the first report on molecular detection and identification of *Leishmania* (*Leishmania*) *infantum chagasi* in a naturally infected domestic cat (*Felis catus*) from Belem, Pará, Brazil. This report suggests that autochthonous leishmaniasis with cats acting as a secondary reservoirs of *Leishmania* (*L.*) *infantum chagasi* is occurring in an important urban area in eastern Amazon. 

## Case presentation

This study was approved by the Animal Use Ethics Committee (CEUA) of the Federal University of Pará (UFPA), under protocol number CEUA-UFPA 20220602. 

**Figure 1.**
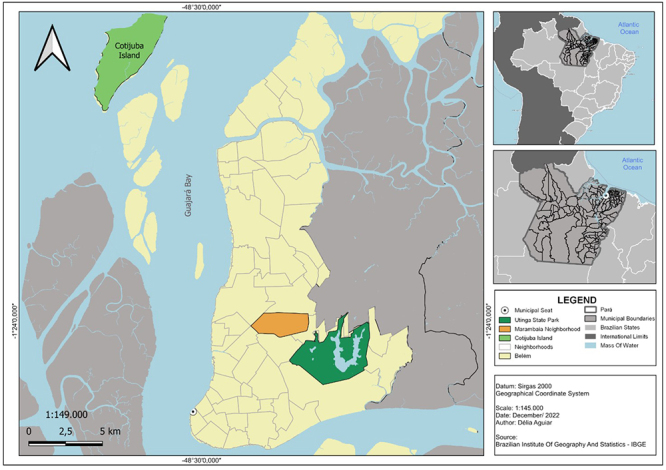
Location of the occurrence of autochthonous feline leishmaniasis described in this study.

In October 2019, a stray cat, approximately 3 years old, was taken to a veterinary service by a cat lover who had been feeding it for about 2 years at the door of his house, which is located in the COAB complex in Marambaia neighbourhood in Belém, the capital of Pará state, Brazil (Figure 1). The person indicated that the feline had an ear lesion for about 6 months and that he sought veterinary evaluation because new cutaneous lesions were appearing in other parts of the cat's body. Then, the animal was sent to the municipal zoonoses center in Belém for a full evaluation by another veterinarian, who collected biological material (blood, serum and aspirate from the lesion) for diagnostic tests.

**Figure 2.**
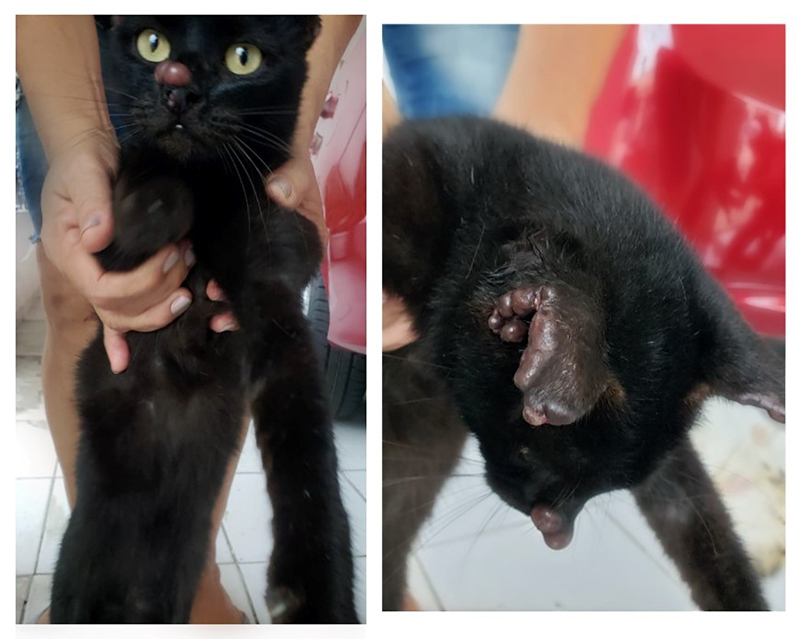
Skin erythematous, edematous, and non-painful papular lesions in a stray cat from an urban area in eastern Amazon.

On physical examination, lymphadenopathy and erythematous, edematous and non-painful papular lesions around the edges of both ears, the snout, and the dewclaw of the left hind limb were observed in the cat (Figure 2). A complete blood count as well as biochemistry and serology tests for visceral leishmaniasis (VL) were performed using enzyme-linked immunosorbent assays (ELISAs), and indirect immunofluorescence assays (IFAs) were performed following the recommendations of the manufacturer (kit ELISA S7 Biogene and kit Biomanguinhos/Fiocruz Brasil). Cytology was performed using fine-needle aspiration cytology (FNAC) and a histopathologic analysis was conducted for tissue examination.

In order to detect *Leishmania* DNA, polymerase chain reactions (PCR) targeting the small subunit ribosomal RNA (SSU rDNA) and kinetoplastid DNA (*kDNA*) genes of the parasite were performed according to Uliana et al. [19] and Francino et al. [20], respectively. Sequencing of the amplicon we obtained was performed in an ABI 3500XL automated DNA analyzer (Applied Biosystems™). Additionally, PCRs were also conducted in order to detect any other infection by haemoparasites (*Ehrlichia* spp., *Cytauxzoon felis*, *Anaplasma* spp. and *Mycoplasma* spp.) or viruses (Feline leukemia virus - FELV, and feline immunodeficiency virus - FIV). A nested PCR amplicon was sequenced in both directions, forward and reverse, using an ABI 3500XL DNA analyzer (Applied Biosystems™). BioEdit software [21], was used to assemble and align sequences. Phylogenetic analysis was carried out through Bayesian Inference (BI) as implemented in MrBayes 3.2.6 [22], assuming a Jukes-Cantor (JC) model of nucleotide substitution, which was selected as the most appropriate model of evolution by ModelFinder as implemented in IQ-Tree 2.1.3 [23], under the Bayesian Information Criterion (BIC). Two parallel runs of four simultaneous Markov Chain Monte Carlo (MCMC) searches for 2 million generations each were performed, sampling one tree every 100 generations, and the results of the first 5000 trees as burn-in were discarded. The remaining trees were used by MrBayes to estimate the posterior probability of each node in our phylogenetic reconstruction. In addition to the sequence obtained here, the ingroup included seven partial SSU rDNA sequences of *Leishmania* species which were retrieved from Genbank. Based on Marcili et al. [24] we used SSU rDNA sequences of *Leptomonas* as an outgroup.

**Figure 3.**
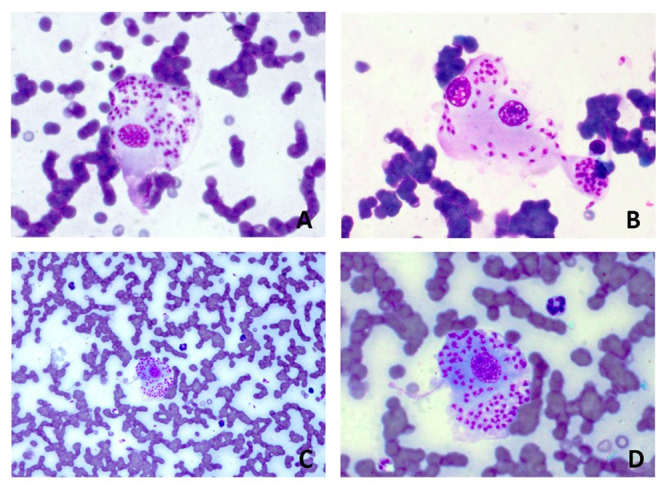
Cytology of the aspirate (panoptic staining) from the nostril of the animal indicating macrophages with amastigotes of *Leishmania* sp. (Magnification **A**, **B** and **D**: 1000×; **C**: 400×).

The blood count indicated only neutrophilia with a regenerative left shift. The serum levels of alkaline phosphatase, creatinine, urea, transaminase glutamic-oxaloacetic (TGO) and glutamate-pyruvate transaminase (GPT) were within the parameters for *Felis catus*. Serological tests for canine visceral leishmaniasis by ELISA and IFA were negative, while histopathological examination indicated severe infectious pyogranulomatous dermatitis caused by *Leishmania* spp. or *Toxoplasma gondii.* However, the cytopathological examination confirmed amastigote forms of *Leishmania* sp. inside macrophages (Figure 3).

**Figure 4.**
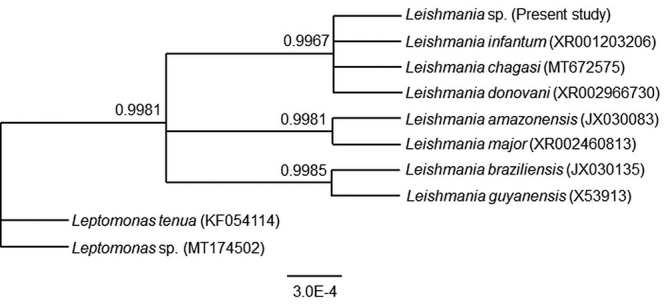
Phylogenetic tree derived from the Bayesian inference based on partial SSU rDNA of *Leishmania*. Bayesian posterior probability values are given above the branch nodes. The scale bar indicates the number of substitutions per nucleotide site.

Molecular detection of hemoparasites and viruses were all negative. There was no detection of *L. infantum kDNA*, but detection of SSU rDNA of *Leishmania* spp. was positive. Excluding primers, a database with 403 nucleotides of the SSU rDNA was generated for most taxa, except for *Leishmania chagasi*, which had only 398. According to the topology found (Figure 4) in group splits in three clades: A (sequence of this study, *Leishmania infantum*, *Leishmania chagasi*, *Leishmania donovani*), B (*Leishmania amazonensis*, *Leishmania major*) and C (*Leishmania braziliensis*, *Leishmania guyanensis*). Sequences within each group showed 100% identity. The clade A sequences differed from the rest by presenting a thymine instead of a cytosine in position 250, while the clade B sequences in position 17 presented an adenine instead of a guanine, as was observed in the others. Finally, the clade C sequences in position 10 presented a thymine instead of the cytosine seen in the other sequences. 

## Discussion

Although we did not seek to evaluate the efficiency of FeL detection methods, the negative serology versus positive PCR, as we observed here, corroborates Fernandez-Gallego et al. [4], who suggests that a combination of diagnostic tests may be needed for definitive diagnosis. In fact, serology may not be enough to reach a diagnosis in negative or low positive cases [25]. Additionally, it is possible that cats behave similarly to dogs in the two scenarios described by Quinell et al. [26]. In the first one, not all infected dogs are expected to be seropositive; there is known to be a significant prepatent period before seroconversion [27, 28], a number of infected dogs may never convert [29, 30], and dogs may revert to seronegative but remain parasite positive. In the second one, it is difficult to discriminate between seropositive and seronegative dogs; bimodal distributions of antibody titers that would identify a distinct population of seropositive animals are rarely seen, and different tests rarely agree on the proportion of positives in a sample [31].

A general problem with *Leishmania* serology is that the frequency distributions of antibody titers for infected and uninfected animals show considerable overlap, regardless of the serological test used [26]. Given that the immunological response from cats can be different from that of dogs and due to the lack of specific validation for cats, the same cut-off values for antibody titers used for testing dogs are applied for testing cats. As a result, the serological tests may not reflect the actual infection status in felines [32, 33]. 

Although PCR is one of the most sensitive methods for leishmaniasis diagnosis, it is noteworthy that molecular detection of *Leishmania* DNA occurs more frequently in cats with reduced antibody titers [32, 34, 35]. In our study we used two different PCR protocols, both targeting genes with a large number of copies in the parasite genome. However, genetic material was detected only in the protocol designed to amplify a SSU rDNA fragment of *Leishmania* spp. through a nested PCR, which is known to present higher sensitivity compared to the detection of *Leishmania infantum kDNA*, which is a simple PCR. In this scenario, our study agrees with Merdekios et al. [36] and Gow et al. [37], who claim that, in order to reduce the number of false negative results, molecular diagnosis of leishmaniasis requires simultaneous use of more than one protocol. 

Regarding identification of the parasite detected in this study, the nucleotide sequence of the amplicon we obtained was consistently grouped in a clade that included *L. infantum*, *Leishmania donovani* and *Leishmania chagasi*. Mauricio et al. [38], suggested that *L.* (*L.*) *chagasi is* synonymous with *L.* (*L*.) *infantum* and therefore proposed that *L*. (*L*.) *chagasi* should not be considered as a valid species. On the other hand, Silveira and Corbett [39] evaluated all available knowledge concerning the eco-epidemiology of *L.* (*L.*) *chagasi* in the Brazilian Amazon, especially in regard to the sylvatic habits of its phlebotomine sandfly vector, *Lutzomyia longipalpis*, and its vertebrate reservoir, the wild fox *Cerdocyon thous*, with the aim of showing that *L*. (*L*.) *chagasi* cannot be neglected in the parasitological investigation of VL in the New World. However, both Silveira and Corbett [39] and Kuhls et al. [40], agree that the name *L*. (*L*.) *infantum chagasi* should be used. Finally, since *L. donovani* is prevalent in East-Africa, India, and parts of the Middle East but not in the New World [41], the taxon in our study was identified as *Leishmania* (*Leishmania*) *infantum chagasi*, the species that is commonly isolated in patients with VL. 

To the best of the authors’ knowledge, this report describes the first case of natural infection by *Leishmania* (*Leishmania*) *infantum chagasi* in a domestic cat (*Felis catus*) in Belém, Pará, Brazil, which is an important urban area in eastern Amazon. Indeed, this is the second confirmed FeL case in Belém. Carneiro et al. [18] reported the first one, which was caused by *Leishmania* (*Leishmania*) *amazonensis*. Another FeL case in Pará state, without identification of the *Leishmania* species, was reported by Mello, in 1940, in a locality 30 km away from Belém. 

The low number of cases of FeL in Belém may be a reflex of greater resistance to the disease due to a more effective cellular immune response in cats than in dogs [11, 42]. Some studies conducted in cats indicate that infection by hemoparasites as well as FIV and FELV can facilitate *Leishmania* infection [4, 10]; however, no infection by hemoparasites, FIV or FELV was detected in the present study, suggesting no immunocompromise in the animal. A meta-analysis indicated that the prevalence of dogs and cats in endemic areas is similar and that the differences may be related to the greater number of studies conducted in dogs than in cats [43]. At any rate, the number of FeL cases in Belém seems to be consistent with the epidemiological status of this municipality, which is an area of sporadic transmission (the average number of cases of VL over the last 5 years is lower than 2.4), although it has an intense migratory flow with municipalities considered areas of moderate (average of cases of VL in the last five years is greater than 2.4 and lower than 4.4) and intense (average of cases of VL in the last five years is greater than 4.4) transmission [44]. 

It is noteworthy that Belém is part of a metropolitan region (Região Metropolitana de Belém - RMB) which has a human population estimated at 2.275.032 and a feline population estimated at nearly 22 million, for which epidemiological data on leishmaniasis are scarce. An exception is the Oliveira et al. [45] study, which analyzed the prevalence of anti-*Leishmania* spp. antibodies in domestic cats in Belém, finding 4.06% (18/443) positive reactions in the indirect immunofluorescence assay (IFA). Considering all issues related to serological tests treated in the present study it is likely that the real rate of *Leishmania* spp. infection in domestic cats in Belém is higher. As an example of this scenario, of the 1,443 dogs examined by Coura-Vital et al. [46], 15.9% were seropositive in at least one ELISA, whereas PCR-RFLP revealed that 24.7% of them were positive based on detection of *L. infantum* DNA.

Since FeL seroprevalence in Belém is similar to that observed in endemic areas in southeastern Brazil [32, 47] the role of the domestic cat in VL epidemiology in Belém should not be neglected. Thus, in addition to dogs, our study points to domestic cats as a potential secondary reservoir host of *L.* (*L.*) *infantum chagasi*, as well as corroborating the hypothesis of adaptation of this parasite to alternative vectors [48], which may be involved in VL urbanization. To the best of our knowledge, *Lutzomya longipalpis*, considered the main vector of the VL, could not be found in Belém, additionally, Lainson and Rangel [48], suggests *Lu. longipalpis* is primordially a sylvatic species and that it can still be captured in remote primary forest that is far from human habitation. Remarkably, the RMB has a total of 154 forest fragments and four urban parks with areas varying from 1 to 1,200 hectares, where the phlebotomine sand fly fauna currently presents 22 known species, with at least six being of epidemiological interest in the potential transmission of five leishmaniasis agents [49]. Thus, the real vector of causative agent of VL has not been elucidated.

## Conclusion

Although our study does not contribute to an understanding of the risk factors associated with FeL in Belém, it does contribute towards expanding knowledge about the elements that make up the chain of transmission of VL. Thus, in the epidemiological context, together with that reported by Carneiro et al. [18] the case presented here corroborates the literature in which domestic cats can play a role as secondary reservoirs hosts for *Leishmania* spp. in urban areas. Additionally, our report highlights the important changes that have occurred in the transmission pattern of VL, which initially had an eminently rural character, but which in recent years has been expanding into medium and large urban areas [44]. In general, this study reinforces the need for active surveillance of FeL in urban areas, which is in line with the measures recommended by the Brazilian Ministry of Health aiming to avoid or minimize the problems related to this disease in those areas [44]. Finally, our study suggests that more research should be developed among cat populations, especially in countries of endemic importance such as Brazil. 
